# Chronic mercury exposure in Late Neolithic/Chalcolithic populations in Portugal from the cultural use of cinnabar

**DOI:** 10.1038/srep14679

**Published:** 2015-10-01

**Authors:** Steven D. Emslie, Rebecka Brasso, William P. Patterson, António Carlos Valera, Ashley McKenzie, Ana Maria Silva, James D. Gleason, Joel D. Blum

**Affiliations:** 1 Department of Biology and Marine Biology, University of North Carolina Wilmington, 601 S. College Rd., Wilmington, NC 28403; 2 Biology Department, Southeast Missouri State University, One University Plaza, Cape Girardeau, MO 63701; 3 Saskatchewan Isotope Laboratory, 114 Science Place, Saskatoon SK S7N5E2, Canada; 4 Archaeological Research Group (NIA – ERA Arqueologia) and ICArEHB center, Calçada de Santa Catarina, 9 C 1495 – 705 Cruz Quebrada, Portugal; 5 Department of Life Science, University of Coimbra, 3000 – 056 Coimbra, Portugal, UNIARQ; 6 Department of Earth and Environmental Sciences, University of Michigan, 1100 North University Ave, Ann Arbor, MI 48109.

## Abstract

Cinnabar is a natural mercury sulfide (HgS) mineral of volcanic or hydrothermal origin that is found worldwide. It has been mined prehistorically and historically in China, Japan, Europe, and the Americas to extract metallic mercury (Hg^0^) for use in metallurgy, as a medicinal, a preservative, and as a red pigment for body paint and ceramics. Processing cinnabar via combustion releases Hg^0^ vapor that can be toxic if inhaled. Mercury from cinnabar can also be absorbed through the gut and skin, where it can accumulate in organs and bone. Here, we report moderate to high levels of total mercury (THg) in human bone from three Late Neolithic/Chalcolithic (5400–4100 B.P.) sites in southern Portugal that were likely caused by cultural use of cinnabar. We use light stable isotope and Hg stable isotope tracking to test three hypotheses on the origin of mercury in this prehistoric human bone. We traced Hg in two individuals to cinnabar deposits near Almadén, Spain, and conclude that use of this mineral likely caused mild to severe mercury poisoning in the prehistoric population. Our methods have applications to bioarchaeological investigations worldwide, and for tracking trade routes and mobility of prehistoric populations where cinnabar use is documented.

Perdigões is a Neolithic/Chalcolithic ditched enclosure site near Évora, south-central Portugal (Fig. 1) that was an important gathering place for over 1000 years (3400–2100 B.C.). The site functioned for ceremonial gatherings and for deposition of human and animal remains and offerings, often with ochre and/or cinnabar in association^1^,^2^; it also served as a celestial calendar^3^,^4^. Ongoing investigations at this site since 1997 have resulted in a multinational research program, the Global Program of Archaeological Research of Perdigões, to test hypotheses on the use and function of this site. One of the main hypotheses under investigation, referred to as the ‘mobility’ hypothesis, is that Perdigões was used by diverse groups from distant as well as local populations in Iberia. Preliminary analysis of strontium isotopes from human teeth supports this hypothesis^2^. Our initial objective was to determine if variation in light stable isotopes values (δ^15^N and δ^13^C) in human bone, which reflect diet (trophic level, plus marine versus terrestrial diets^5^,^6^) and latitude, as well as photosynthetic pathways of plant food^7^,^8^,^9^,^10^,^11^,^12^, would also support this ‘mobility’ hypothesis. Total mercury (THg) analysis of the bone was included as part of this study as significant variation in mercury exposure among individuals, presumably caused by differences in primary diets^13^, could also provide suitable data to test this hypothesis.

While initially it was expected that mercury exposure would be minimal in the Perdigões population, our results on bone from 45 individuals from three Neolithic/Chalcolithic sites with group burial features were surprising. Most individuals had moderate to high levels of THg in their bone (range 0.06–188.9 μg/g with >10 μg/g in 31 individuals). As no previous study had analyzed THg in Neolithic/Chalcolithic human remains, the unexpectedly high level of exposure we observed became the main focus of our research. Here, we use a combination of light stable isotope (δ^15^N and δ^13^C), THg analyses of additional bone and soil, and Hg isotope analysis to test three hypotheses on the source of this mercury and its potential impact on the health of this prehistoric population.

## Results

We analyzed a total of 37 samples of human bone, five animal bones, and eight soil samples from Perdigões. We also analyzed 11 human bone samples from two other Neolithic/Chalcolithic sites in southern Portugal: Sobreira de Cima (n = 5)^14^ where cinnabar also was identified with human burials^15^, and Monte Canelas I (n = 6 from three individuals) where no cinnabar was found associated with the burials^16^. Sobreira de Cima is a necropolis where five tombs containing hundreds of individuals in various states of preservation and articulation were excavated; ochre and cinnabar were found in all of these features and, in Tombs 2 and 3, these minerals were in sufficient quantities to form ‘red beds’ in the deposits^14^. Radiocarbon dates from four tombs range from 4080–4670 B.P. We choose five femora from five different stratigraphic units (UE11–15; Table S1) from Tomb I for analysis; cinnabar was identified with the burials from this tomb^15^, though no soil samples remain from the excavations for analysis.

Monte Canelas I is a hypogeum that contained over 6000 human bones from at least 150 individuals and dates to approximately 4400 B.P^16^. While most of these remains were scattered fragments, five primary burials were uncovered from the lower burial level of the hypogeum^16^. Of these, three were from adults: a middle-aged male (270), an old female (337), and a young adult female (342). Two bones from each of these individuals, a humerus and tibia, were analyzed for THg to help understand intra-skeletal variation. In all three individuals, the humerus had consistently higher mean ± SD THg (4.9 ± 1.9) than in the tibia (2.8 ± 1.0; Table S1).

The 37 human bones from Perdigões date from 3840 to 4430 B.P^4^. and include juveniles and adults of both sexes (Table S1). These remains were recovered from five burial features: Pits 7, 11, and 16, and Tombs I and II (Table S1). These features and the context of the burials within them are described by Valera *et al.*^2^ and Valera^3^; only Tombs I and II had ochre and cinnabar in association with the burials. The mean ± SD THg concentrations in known adult males (n = 7) and females (n = 8), respectively, were 67.1 ± 34.5 μg/g and 70.4 ± 69.0 μg/g with no significant difference between the two groups (*t* test, t = −0.117, *p *= 0.909; Table S1). Five juveniles, however, had a significantly lower mean THg concentration at 10.2 ± 17.3 μg/g than male and female adults combined (t < −3.53, *p *< 0.045). This latter result was expected as juveniles would have had less exposure time in life to accumulate mercury in their tissues, either through diet or other pathways. However, one juvenile from Sobreira de Cima had a very high THg value at 133.1 μg/g, indicating considerable exposure by this individual early in life.

We have identified three potential sources of the prehistoric mercury exposure in the human bone at Perdigões: diagenetic processes in the soil, dietary ingestion, and cultural use of cinnabar. Here, we provide data that test each of these hypotheses to fully understand how mercury was impacting the prehistoric populations.

### Diagenetic Hypothesis

When first encountering high levels of THg in the human bone from Perdigões, our initial reaction was that soil contamination was the primary cause. Mercury could be absorbed or intrude into the bone after burial by contact with contaminated soil. We investigated this possibility first as cinnabar, a natural mercury sulfide (HgS) that was used prehistorically in Iberia^17^,^18^ and ochre were found sprinkled over some of the human remains at Perdigões and was therefore an obvious source of contamination. Although cinnabar has very low solubility in soil water^19^, dissolution of HgS can occur in oxidized fluvial environments^20^,^21^ and in rare circumstances may then penetrate into bone pores and/or dentinal tubules in teeth^22^,^23^. In addition, tiny particles of cinnabar may have become embedded within the bone matrix, also causing the high THg concentrations. Thus, we chose to investigate these possibilities to further verify whether the mercury in the human bone was deposited there *in vivo* versus after burial.

Because all human remains recovered from Perdigões to date were excavated prior to our research, no *in situ* soil could be analyzed from the tombs. However, we were able to extract a small amount (<1 g) of soil still attached to the interior shaft of two of the 37 human bones from Tomb II atrium and chamber, respectively, and from one pig (*Sus* sp.) bone recovered at the site (Table S1). Results on the human bone indicated that the soil contained either much higher (>50 μg/g) or lower (13.5 μg/g) THg than the bone from which it was extracted (Table 1 and Table S1). The soil attached to the pig bone also had a higher THg concentration (5.8 μg/g) than the bone itself (0.01 μg/g; Table 1). Thus, there are no emerging patterns in soil versus associated bone THg concentrations that might indicate diagenetic contamination. In addition, our analysis of the soil taken from the surface at the location of Tombs I and II and additional animal bones chosen at random across the site indicate lower levels of mercury in most of the soil and very low levels in animal bone compared to the human bones (Table 1), providing evidence that diagenetic processes were not responsible for deposition of mercury in human bone. The consistently different levels of THg in the humerus versus the tibia in three individuals at Monte Canelas I also are in accordance with *in vivo* deposition of mercury, with variation in skeletal elements and bone tissue types (trabecular versus cortical bone) based on blood flow and bone remodeling rates^24^. These results are similar to findings by Rasmussen *et al.*^25^,^26^,^27^ and Ávila *et al.*^28^ who also examined mercury contamination in bone from soil.

Scanning Electron Microscopy and Energy Dispersive X-ray Spectroscopy (SEM-EDS) were performed on three human bones from Perdigões to examine the inner lattice structure and pore spaces of the compact bone and to quantify the elemental composition of the bone for any indication of cinnabar and/or intrusion of soil particles. We selected bone samples that had moderate to high THg for this analysis (40.0–137.4 μg/g; Table S1). The image for one of these samples (Fig. 2) indicate no apparent incursion of soil into the inner bone and is representative of all three samples in displaying a clean and well-preserved lattice. Further, the elemental analysis produced no detectable levels of Hg in the pore space or on the lattice in all three samples. These results support our conclusion that soil contamination is not the primary source of the high levels of THg in the human bone at Perdigões, though additional testing of human and animal bone and soil from future burial pits or tombs at Perdigões and other Neolithic/Chalcolithic sites where cinnabar was known to have been used is warranted.

### Dietary Hypothesis

Our second hypothesis posits that mercury exposure in the prehistoric population was the result of dietary consumption of piscivorous marine and freshwater fish with elevated concentrations of methylmercury (CH_3_Hg), the most common means of mercury exposure in humans today^13^,^29^. As CH_3_Hg levels in consumer tissues usually correlate with trophic level of diet reflected by δ^15^N, we subsequently extracted collagen from all 45 bone samples for δ^15^N analysis (Table S1, but using only one bone from each of the burials from Monte Canelas I). Of these, ten samples were preserved well enough to produce an atomic C:N ratio between 2.9–3.6 that indicates reliable results^30^ (Table S2). These ten samples showed a weak negative relationship between δ^15^N and THg (Fig. 3; R^2 ^= 0.0384), opposite of what is expected if dietary CH_3_Hg was the source of the THg in the bone.

To further investigate the effect of diet on THg concentrations, we analyzed CH_3_Hg concentrations in three bone samples (Perdigões, n = 2 and Sobreira de Cima, n = 1) to determine the proportion of THg that was CH_3_Hg. Methylmercury concentrations in these three samples ranged from 0.00137 to 0.00243 μg/g (all <0.05% of THg; Table S3), indicating that nearly all of the mercury in the bone is in the inorganic form. It should be noted that the proportion of CH_3_Hg may have been higher *in vivo* as it can demethylate via microbial processes in the soil environment. Thus, the concentration of CH_3_Hg in bone presented here represents only the organic mercury remaining in the bone at the time of analysis. These results, however, indicate that the high THg in the human bone from Perdigões, and in two cases Sobreira de Cima, are not the result of ingestion of methylmercury in food.

### Cultural Use of Cinnabar Hypothesis

Our third hypothesis regarding the source of mercury exposure is from the cultural use of cinnabar that resulted in accidental ingestion, inhalation, or absorption through the skin. Physiological pathways for mercury poisoning from cinnabar are reviewed by Liu *et al.*^19^ While raw cinnabar is much less toxic than CH_3_Hg, long term exposure, accidental ingestion, and absorption through the skin and gut can lead to chronic effects with deposition of mercury occurring primarily in the kidney, liver, and brain. Use of cinnabar as a pigment for pottery, for burial offerings, body paint or tattoos, could result in repeated exposure and accidental ingestion. If used as a medicinal, additional exposure would occur. Burning cinnabar releases highly toxic mercury vapor that can cause immediate effects, including death^19^. Moreover, all of these pathways were probably more limited in juveniles, with less exposure time in life, and may account for the low levels of mercury found in most of their bones here.

**Figure 1:**
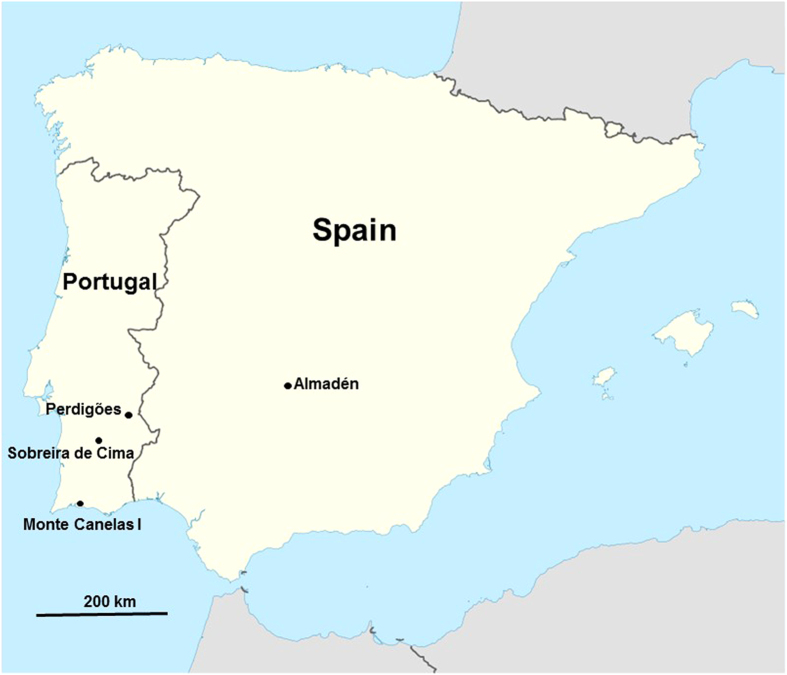
Map of Iberia with locations of major sites discussed in the text. Modified from an outline map available online (https://commons.wikimedia.org/wiki/File:Iberian_Peninsula_location_map.svg) that is licensed under the Attribution-ShareAlike 3.0 Unported license. The license terms can be found on the following link: https://creativecommons.org/licenses/by-sa/3.0/.

**Figure 2:**
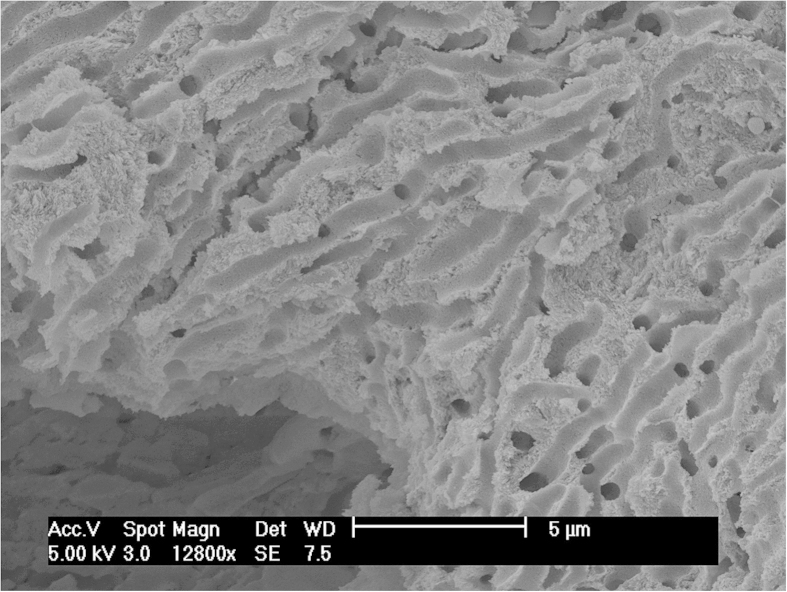
SEM image of the inner compact bone. The image is from Perdigões sample 3934 from Tomb II atrium, distal right humerus of an adult female that had 137.41 μg/g THg. No soil or cinnabar particles are visible in the inner bone and x-ray microanalysis indicated undetectable levels of Hg.

**Figure 3:**
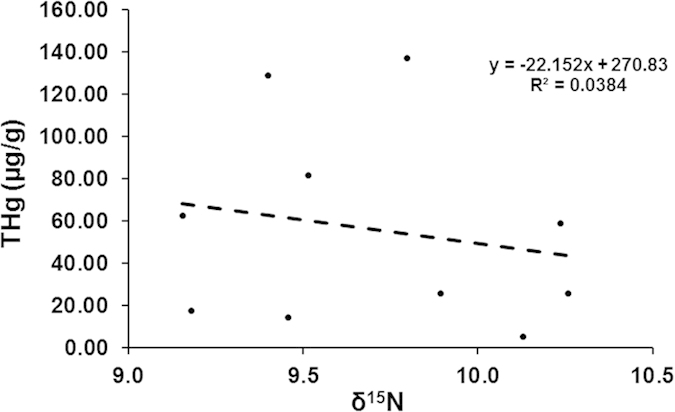
THg versus δ^15^N analysis. Values plotted are from eight human bone samples at Perdigões and two from Sobreira de Cima with collagen C:N ratios between 2.9–3.6. The higher values of THg with lower δ^15^N are opposite of expectations if methylmercury (CH_3_Hg) was the source of mercury in the bone.

**Figure 4:**
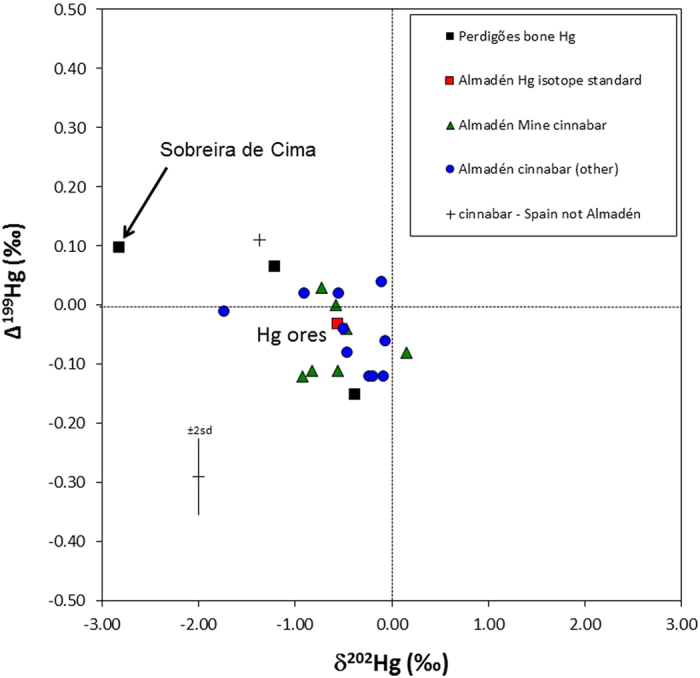
Hg isotopic compositions. Values (δ^202^Hg versus Δ^199^Hg) plotted include three Neolithic human bone samples (two from Perdigões, one from Sobreira de Cima) compared to cinnabar values from the Almadén mine, Spain. Note the isotopic similarity of the Perdigões samples to Almadén cinnabar, while the Sobreira de Cima sample has a much lower δ^202^Hg value (−2.83%0), outside the range of known values for cinnabar Hg.

One of the largest natural sources of mercury in the world is located at cinnabar mines near Almadén, Spain^31^,^32^,^33^, approximately 300 km ENE of Perdigões (Fig. 1). This mine was used by the Romans to extract mercury from the ores; laborers here often suffered high mortality as a result of these extraction processes^34^. Previous research on Pb isotope tracking has demonstrated that cinnabar was being mined here by the early Neolithic (6^th^ millennium B.C. or ~7300 B.P.)^18^, supporting archaeological evidence for its use in the Neolithic of Spain^17^. Similarly, Hg isotopes have been used to successfully track mercury sources in ecological contexts as well as in one archaeological study where cinnabar mining in Peru caused high levels of Hg to be deposited in lake sediment^35^,^36^. Accordingly, we analyzed the same three samples of human bone used for CH_3_Hg analysis to characterize their Hg isotopic composition (δ^202^Hg and Δ^199^Hg). Results from the three bone samples indicate significant isotopic variation, with δ^202^Hg values ranging from −2.83%0 to −0.38%0, and Δ^199^Hg values ranging from −0.15%0 to +0.10%0 (Fig. 4; Tables S4–S5). These values demonstrate both mass dependent and mass independent fractionation of Hg isotopes relative to the NIST3133 standard. Significantly, the two Perdigões bone samples fall within the range of values previously determined for cinnabar ore from the Almadén region^33^ while the bone sample from Sobreira de Cima falls outside of this range (Fig. 4). The Hg isotopic compositions recorded at Perdigões are consistent with inheritance of high levels of inorganic Hg from Almadén cinnabar during the lifetimes of these individuals. The variation in the bone sample from Sobreira de Cima may reflect inheritance from another inorganic Hg source, possibly cinnabar, which has not yet been characterized for Hg isotopes.

## Discussion

Only a few studies have previously investigated THg in archaeological human bone. Yamada *et al.*^36^ found high levels of THg (>100 μg/g) in 6–7^th^ and 12–17^th^ century burials in Japan that were equivalent, and in some cases much greater, than levels found here. The authors attributed these high levels not to Hg in soil, where levels were low or undetectable, but to use of body paints containing Hg. Research by Rasmussen *et al.*^24^ on medieval human remains also documented Hg levels in bone ranging from <0.02 to 2.16 μg/g, attributed to the use of red ink and medicines containing mercury by monks. Cervini-Silva *et al.*^22^ and Ávila *et al.*^28^ applied high-resolution microdiffraction and x-ray diffraction analyses, respectively, to assess mercury presence in archaeological human bone in Mesoamerica where cinnabar was used as a preservative. While extensive use of cinnabar applied over bone can result in adsorption via fungal growth^22^, and a red stain on the bone surface^23^, Ávila *et al.*^28^ found that Hg in bone hydroxyapatite was likely caused by inhalation of Hg^0^ vapor and cinnabar dust in life or absorption through the skin.

Given that cinnabar was the likely source of most of the THg in human bone at Perdigões, the question remains as to whether this exposure was high enough to have caused mercury poisoning. Studies of bone as a biomarker for mercury exposure are rare and suggest that deposition of mercury into bone is minimal compared to other tissues^25^,^38^,^39^,^40^. One post-mortem study of workers at a smelter in Sweden who were exposed to inorganic Hg for more than ten years had <0.1 μg/g of THg in their bone^40^. An autopsy analysis of human subjects in Spain revealed detectable levels of mercury in liver and kidney tissue (0.14 and 0.25 μg/g, respectively) but <0.05 μg/g in bone^39^. Two studies on non-human mammalian subjects had similar results with bone having the lowest THg levels compared to other tissues^41^,^42^. It follows then that inorganic Hg inhaled, ingested or absorbed through the skin or gut from cinnabar will be deposited in bone in lesser amounts than in other tissues such as the kidney. If so, the levels of THg we observed in archaeological human bone would correspond with levels at least 5–10 times higher in the corresponding soft tissues. Thus, human bone with >10 μg/g THg likely represents a severe and chronic exposure that affected health and mortality. For comparison, THg in hair of 10 μg/g is considered to be the tolerance level for mercury exposure today, impacting motor function and vision^13^. However, no such guidelines exist for bone. Numerous other adverse effects to health are known to occur with both inhalation of Hg^0^ vapor and ingestion of Hg^0^ or Hg(II)^43^ and both of these pathways were probably occurring with prehistoric humans using cinnabar^28^.

Because cinnabar occurs in natural mineral deposits worldwide, with major sources in Spain, Europe, and the Americas^20^, prehistoric use of these deposits would have increased Hg availability both locally and with trade or transport to other regions. The impact this may have had on the prehistoric population at Perdigões and at other Neolithic/Chalcolithic sites in Iberia regarding their health, social structure and cultural behavior is unknown, but likely was significant and is worthy of additional investigation. Our application of Hg isotope tracking in archaeological human bone also provides a new tool for investigating prehistoric mobility and the trade and transport of cinnabar from specific geological deposits where it was mined.

## Methods

### Total mercury analyses

Human bone samples were prepared by removing a small section of compact bone (2–3 cm wide) from the shaft of each sampled long bone. Though none of our samples had obvious cinnabar or red staining on the surface, each sample was thoroughly washed under running distilled water. After drying, the outer surface of each bone was removed using a 3/32 inch carbide bur and an NSK Ultimate XL micromotor. The drill operator wore latex gloves and a dust mask to prevent contamination. The tools and surface area were cleaned with ethanol after each sample was processed. Exposed inner bone was sampled with the drill and the powdered bone captured on a clean piece of aluminum foil, then transferred to a clean vial prior to analysis. For Hg isotope analysis of larger bone samples, 3–5 g of compact bone was prepared as described above, with all exposed surfaces (interior and exterior) removed using the drill. The bone was then rewashed in distilled water and pulverized to powder using a ceramic mortar and pestle. The mortar and pestle were cleaned after each use with ethanol and then rinsed with distilled water.

Soil samples were collected and stored in sterile plastic bags. Samples were freeze-dried to remove all moisture and then sieved through a 500 μm screen onto a clean piece of aluminum foil to remove coarse grains and rock; the sieve was washed with distilled water and ethanol after each sample. THg was measured with a Tri-Cell Direct Mercury Analyzer (DMA-80). Each set of 20 samples was preceded and followed by two method blanks, a sample blank, and two samples each of standard reference material (DORM-4 and DOLT-5 from fish protein and dogfish liver, respectively, and certified by the National Research Council, Canada).

### SEM-EDS analysis

Three samples of inner compact bone, cleaned and prepared for THg analysis as described above, were mounted on aluminum stubs with carbon tape and coated with 10 nm of platinum-palladium (80:20) with a Cressington 208 HR sputter coater. The samples were imaged with the Philips XL30S FEG scanning electron microscope in secondary electron mode to choose areas for analysis. Once the areas of interests were chosen, each sample was examined utilizing the Phoenix EDAX non-dispersive x-ray microanalysis mode operated at 25 kV accelerating voltage. The minimum effective detection limit for Hg was 0.1%.

### Light stable isotope analyses

Collagen was extracted from the powdered bone following published procedures^44^. First, the sample was demineralized in 10% HCl at room temperature until the reaction was complete. Residues were neutralized with deionized water by centrifugation. Next, humic acid was removed by treating the samples with 0.125 M NaOH for 20 hrs at room temperature. Samples were rinsed again with deionized water to neutral pH, dried overnight at 60 °C and homogenized. Samples were loaded into tin cups and processed using a Thermo Finnigan Flash 1112 EA coupled to a Thermo Finnigan Delta Plus XL via a Conflo III. Resulting carbon isotope ratios were corrected for ^17^O contribution using the Craig correction, and reported in per mil notation relative to the VPDB scale. Nitrogen isotope ratios are reported in per mil notation relative to AIR. Carbon data were calibrated against the international standards L-SVEC (δ^13^C = −46.6%0 VPDB) and IAEA-CH6 (δ^13^C = −10.45%0 VPDB). Results with C:N ratios outside of a reliable range for well-preserved collagen (2.9–3.6)^30^ were disregarded.

### Hg stable isotope analysis

Hg in three bone samples was extracted by using a 2-stage furnace combustion system. Ceramic boats containing approximately 0.02 g of bone were loaded into the first combustion oven which was heated to 750 **°**C over the course of six hours to release the Hg in the samples as Hg^0^. Hg^0^ released from the bone was transported through the second furnace (1000 °C) using Hg-free oxygen as a carrier gas, and trapped in a highly oxidizing solution of 1% KMnO_4_ in 10% trace metal grade H_2_SO_4_. The Hg^2+^ in this solution was then neutralized using 5% hydroxylamine and reduced back to Hg^0^ using SnCl_2_ during a purge and trap separation process to concentrate the sample and remove any remaining combustion product matrix from the solution. Recoveries from the combustions and purge and trap for the three samples ranged from 83–116%; recovery was 95% for the standard reference material (DORM4, n = 1).

High precision isotope ratios were obtained with a multi-collector ICP-MS at the University of Michigan by continuous flow cold vapor generation using a Tl internal standard combined with standard-sample-standard bracketing to correct for instrumental mass bias^45^. Reported Hg isotope ratios use standard delta notation (in per mil units or %0), relative to the NIST SRM-3133 Hg internal standard^46^. ^202^Hg/^198^Hg ratios are reported as δ^202^Hg, which describes mass dependent fractionation (MDF). Capital delta notation (∆^199^Hg and ∆^201^Hg) is employed to express the deviation from mass dependence, or mass independent fractionation (MIF), of the odd isotopes ^199^Hg and ^201^Hg. The ability to distinguish small isotopic differences between samples is illustrated by the long-term precision obtained on the UM-Almadén Hg secondary standard, which is employed as an external standard for all experiments, and has a long-term average (±2 SD) of −0.57 ± 0.08%0 (δ^202^Hg) and −0.02 ± 0.05%0 (∆^199^Hg).

### Statistical analysis

Mean THg values for samples grouped by age and/or sex were compared with a two-tailed *t* test using JMP 11.0 with *p *< 0.05. Linear regression analysis was completed in Excel 2013.

## Additional Information

**How to cite this article**: Emslie, S. D. *et al.* Chronic mercury exposure in Late Neolithic/Chalcolithic populations in Portugal from the cultural use of cinnabar. *Sci. Rep.* **5**, 14679; doi: 10.1038/srep14679 (2015).

## Supplementary Material

Supplementary Information

## Figures and Tables

**Table 1:** THg of animal bone and soil samples.

Samples	Element	Weight (g)	THg (μg/g)
Animal bones			
Pig (*Sus* sp.)	metapodia shaft	0.0177	0.01
Sheep/goat (*Ovis*/*Capra* sp.)	distal right humerus	0.0335	0.01
Pig (*Sus* sp.)	metapodia shaft	0.0244	0.041
Pig (*Sus* sp.)	distal left humerus	0.034	0.01
Soil			
Pit 40, Level 294, Square 3		0.0123	3.42
Profile wall, 30 cm depth		0.011	6.55
Profile wall, 30 cm depth		0.011	8.77
Surface, 1 cm depth, by Tomb I		0.0108	32.21
Surface, 1 cm depth, by Tomb II		0.0125	0.11
Soil attached to *Sus* sp. humerus		0.0057	5.78
Soil inside human bone (Tomb II atrium)		0.016	26.5
Soil inside human bone (Tomb II chamber)		0.013	67.6

THg values in parts per million (μg/g).

Species and skeletal element of animal bones and proveniences of soil samples from Perdigões are given with corresponding THg values in parts per million (μg/g).
