# Supplementary material for: Chronic mercury exposure in Late Neolithic/Chalcolithic populations in Portugal from the cultural use of cinnabar
Source: Sci Rep. 2015 Oct 1;5:14679. doi: 10.1038/srep14679 (PMC4589774; doi:10.1038/srep14679)
Supplement: Supplementary Information [file srep14679-s1.doc]

Chronic mercury exposure in Late Neolithic/Chalcolithic populations in Portugal from the cultural use of cinnabar

Steven D. Emslie, Rebecka Brasso, William P. Patterson, António Carlos Valera, Ashley McKenzie, Ana Maria Silva, James D. Gleason, and Joel D. Blum

**Table S1. THg values of human bone.** Data include 48 human bones (37 from Perdigões, five from Sobreira de Cima, and six from three individuals at Monte Canales I). Samples with multiple values of THg are those that were analyzed more than once. An asterisk (*) indicates those samples used for SEM-EDS analysis; a dagger (^ϯ^) indicates those samples from which soil was extracted from the interior shaft for THg analysis (Table 1).

| **Provenience/Feature** | **Element** | **Age/Sex** | **Weight** | **THg (μg/g)** |
| --- | --- | --- | --- | --- |
| **Burial Pits** |  |  |  |  |
| Pit 7 114 #20 | dist left fibula | Adult F | 0.0204 | 0.26 |
| Pit 11 #76 | prox right humerus | Juv. M | 0.0228 | 0.06 |
| Pit 11 #77 | prox right femur | Juv. M | 0.0303 | 0.06 |
| Pit 11 #78 | right femur | Juv. M | 0.0301 | 0.12/0.11 |
| Pit 16 #90.2 | rib fragment | Adult | 0.0205 | 1.4/2.0 |
| Pit 16 #74.2 | rib fragment | Adult | 0.0214 | 0.41 |
|  |  |  |  |  |
| **Tomb I Chamber** |  |  |  |  |
| F7 #42 97 | femur | Adult | 0.0204 | 18.2/33.1/16.1 |
| B5 #59 97 | right femur | Adult | 0.0068 | 6.4/7.4 |
| #1754 447 302 | femur | Adult | 0.0057 | 17.6 |
| #117 63 | left femur | Adult | 0.0057 | 1.8/4.8 |
| E7 #105 458 169 | left femur | Adult | 0.0064 | 32.2/19.2 |
| #1432, 1058 | dist right humerus | Adult M | 0.0058 | 73.1 |
| #1076, 1109 | dist left humerus | Adult M | 0.0067 | 52.8 |
| F2 #15, UE136, 028 | dist left humerus | Adult M | 0.0023 | 3.4 |
| C4 #42, 221 | dist right humerus | Adult F | 0.0054 | 24.6 |
| #1490, 1094 | dist right humerus | Adult F | 0.0053 | 17.3 |
| F7 #42 UE97 205 | dist left humerus | Adult F | 0.0077 | 9.0 |
| F3 #36 UE173 215 | dist right humerus | Adult | 0.0073 | 75.9 |
|  |  |  |  |  |
| **Tomb II Chamber** |  |  |  |  |
| ^ϯ^Bag 2278 #1823 | prox left femur | Adult | 0.029 | 9.6/11.5 |
| Bag 1236 #429 | right femur | Adult | 0.0301 | 11.8 |
| Bag 2253 #429 | right femur | Adult | 0.0062 | 77.4/48.9/106.4 |
| Bag 1133 #429 | right femur | Adult | 0.009 | 37.8 |
| Bag 1339 #402 | prox left femur | Adult | 0.006 | 65.5 |
| *Bag 2449, 1456, [429] | left humerus | Adult F | 0.0057 | 101.4 |
| Bag 1099 [429] 1823 | left humerus | Adult M | 0.0053 | 59.1 |
| 1076 [429] 1801 | left humerus | Adult M | 0.0053 | 81.8 |
| Bag 2307 1498/1499 [429] | left humerus | Adult M | 0.0057 | 70.6 |
| Bag 1024 [445] | left humerus | Adult M | 0.0053 | 129.0 |
|  |  |  |  |  |
| **Tomb II Atrium** |  |  |  |  |
| Bag 638 #227 | left femur | Adult | 0.0292 | 14.5 |
| Bag 1370 #239 | right femur | Adult | 0.0103 | 63.2 |
| *Bag 3934 [231] 267 | dist right humerus | Adult F | 0.0056 | 137.4 |
| Bag 1154 [452] | dist left humerus | Adult F | 0.0062 | 188.9 |
| *^ϯ^Bag 1259 678 [232] | dist right humerus | Juv. | 0.0054 | 40.0 |
| Bag 2644 [263] 444 | dist left humerus | Adult F | 0.0036 | 84.3 |
| Bag 3955 #108 | left femur | Adult | 0.0052 | 62.6 |
| Bag 3043 #614 | left femur | Adult | 0.0063 | 115.6/106.7/136.6 |
| Bag 2631 #486 | right femur | Juv. | 0.0296 | 10.6 |
|  |  |  |  |  |
| **Sobreira de Cima Tomb I** |  |  |  |  |
| UE11, C3 | prox left femur | Juv. | 0.0052 | 133.1 |
| UE12, B2 | midshaft right femur | Adult | 0.0049 | 7.5 |
| UE13, B1 | midshaft left femur | Adult | 0.0075 | 40.8 |
| UE14, B2 | left femur fragment | Juv. | 0.0052 | 26.2 |
| UE 15, B1/B2/C1 | prox right femur | Juv. | 0.0067 | 53.7 |
|  |  |  |  |  |
| **Hipogeu Monte Canelas I** |  |  |  |  |
| 337 | right humerus shaft | Adult F | 0.0038 | 7.0 |
| 337 | right tibia shaft |  | 0.0042 | 4.0 |
| 342 | right humerus shaft | Adult F | 0.0038 | 3.2 |
| 342 | left tibia dist shaft |  | 0.0051 | 2.2 |
| 270 | right humerus shaft | Adult M | 0.0039 | 4.4 |
| 270 | left tibia dist shaft |  | 0.0045 | 2.3 |

**Table S2. Stable isotope and THg values**. Values of δ^15^N, δ^13^C and THg in μg/g are provided for ten human bones from Perdigões (N = 8) and Sobreira de Cima (N = 2) with C:N ratios between 2.9-3.6 that indicate adequate collagen preservation^30^.

| **Sample** | **δ^13^C** | **δ^15^N** | **C:N** | **THg** |
| --- | --- | --- | --- | --- |
| Perdigões Tomb I, 458, 169 E7, 105 | -25.3 | 9.9 | 3.2 | 25.7 |
| Perdigões Tomb I, 1759, 997, 802 | -24.7 | 9.2 | 3.0 | 17.6 |
| Perdigões Tomb II Chamber S5 (E) | -19.63 | 9.40 | 3.16 | 129.0 |
| Perdigões Tomb II Chamber S10 (J) | -20.11 | 9.51 | 3.39 | 81.8 |
| Perdigões Tomb II Chamber S11 (K) | -19.61 | 10.23 | 3.20 | 59.1 |
| Perdigões Tomb II Atrium S13 (M) | -19.87 | 9.80 | 3.11 | 137.4 |
| Perdigões Tomb II bag 63, 231 #227 | -20.4 | 9.5 | 3.5 | 14.5 |
| Perdigões Tomb II Atrium #108, 3955, 261 | -20.9 | 9.2 | 3.6 | 62.6 |
| Sobreira do Cima Tomb I, UE14, B1, S22 (V) | -20.15 | 10.26 | 3.63 | 26.2 |
| Sobreira do Cima Tomb I, UE12, B2, S23 (X) | -20.58 | 10.13 | 3.34 | 5.7 |

| **Sample** | **Vol. (uL)** | **Peak area** | **Measured**  [**C**](http://en.wikipedia.org/wiki/Carbon)[**H**](http://en.wikipedia.org/wiki/Hydrogen)**_3_**[**Hg**](http://en.wikipedia.org/wiki/Mercury_(element))**^+^ (pg)** | **[**[**C**](http://en.wikipedia.org/wiki/Carbon)[**H**](http://en.wikipedia.org/wiki/Hydrogen)**_3_**[**Hg**](http://en.wikipedia.org/wiki/Mercury_(element))**^+^] in bomb (pg/mL)** | **Total acid in bomb (mL)** | **Total** [**C**](http://en.wikipedia.org/wiki/Carbon)[**H**](http://en.wikipedia.org/wiki/Hydrogen)**_3_**[**Hg**](http://en.wikipedia.org/wiki/Mercury_(element))**^+^ in bomb (pg)** | **Mass in bomb (g)** | **[**[**C**](http://en.wikipedia.org/wiki/Carbon)[**H**](http://en.wikipedia.org/wiki/Hydrogen)**_3_**[**Hg**](http://en.wikipedia.org/wiki/Mercury_(element))**^+^] ug/g** | **THg (ug/g)** | **Est. %** [**C**](http://en.wikipedia.org/wiki/Carbon)[**H**](http://en.wikipedia.org/wiki/Hydrogen)**_3_**[**Hg**](http://en.wikipedia.org/wiki/Mercury_(element))**^+^** |
| --- | --- | --- | --- | --- | --- | --- | --- | --- | --- | --- |
| Sobreira de Cima Tomb I UE12 B2 | 500 | 37 | 3.887 | 7.774 | 5 | 38.871 | 0.0283 | 0.001 | 2.77 | 0.0496 |
| Perdigões Tomb I Chamber #117 63 | 500 | 54 | 5.806 | 11.613 | 5 | 58.065 | 0.0314 | 0.002 | 4.28 | 0.0432 |
| Perdigões Tomb I Chamber B5 #59 97 | 500 | 63 | 6.823 | 13.645 | 5 | 68.226 | 0.028 | 0.002 | 7.53 | 0.0324 |

**Table S3. Results of** [**C**](http://en.wikipedia.org/wiki/Carbon)[**H**](http://en.wikipedia.org/wiki/Hydrogen)**_3_**[**Hg**](http://en.wikipedia.org/wiki/Mercury_(element))**^+^** **analysis.** Three human bone samples from Sobreira de Cima (*N* = 1) and Perdigões (*N* = 2) with total [C](http://en.wikipedia.org/wiki/Carbon)[H](http://en.wikipedia.org/wiki/Hydrogen)_3_[Hg](http://en.wikipedia.org/wiki/Mercury_(element))^+^ (μg/g) and percent of THg as measured in sample “bombs”. Values of THg differ from those for the same samples in Table S2 due to different lab measurements.

| **Sample** | **δ^202^Hg (‰)** | **∆^201^Hg (‰)** | **∆^199^Hg (‰)** |
| --- | --- | --- | --- |
| Sobreira de Cima Tomb I UE12 B2 | -2.83 | 0.02 | 0.10 |
| Perdigões Tomb I Chamber #117 63 | -0.38 | -0.12 | -0.15 |
| Perdigões Tomb I Chamber B5 #59 97 | -1.22 | -0.07 | 0.07 |
|  |  |  |  |
| **Almadén Mine - cinnabar** | 0.15 | -0.12 | -0.08 |
|  | -0.56 | -0.08 | -0.11 |
|  | -0.47 | -0.05 | -0.04 |
|  | -0.92 | -0.03 | -0.12 |
|  | -0.82 | -0.11 | -0.11 |
|  | -0.58 | -0.03 | 0 |
|  | -0.73 | -0.02 | 0.03 |
|  |  |  |  |
| **Almadén cinnabar - other locations** | -0.46 | -0.12 | -0.08 |
|  | -0.50 | 0.01 | -0.04 |
|  | -1.73 | -0.06 | -0.01 |
|  | -0.23 | -0.06 | -0.12 |
|  | -0.19 | -0.07 | -0.12 |
|  | -0.10 | 0.01 | 0.04 |
|  | -0.90 | 0.02 | 0.02 |
|  | -0.55 | -0.01 | 0.02 |
|  | -0.08 | -0.08 | -0.12 |
|  | -0.06 | 0.01 | -0.06 |
|  |  |  |  |
| **Spain cinnabar - not Almadén** | -1.37 | 0.12 | 0.11 |
|  |  |  |  |
| **Elemental Hg ore - various in Spain** | 0.26 | -0.16 | -0.02 |
|  | -0.84 | 0.06 | 0.09 |
|  | -1.36 | 0.15 | 0.03 |

**Table S4. Hg isotope results.**  Hg isotopic compositions of three human bone samples (this study) from Sobreira de Cima (*N* = 1) and Perdigões (*N* = 2) compared to cinnabar ore from the Almadén mine and other locations in Spain^33^.

**Table S5. Hg isotopic compositions of laboratory standards and of Neolithic human bone samples.** Hg composition for three human bone samples from Perdigões (*N* = 1) and Sobreira de Cima (*N* = 2), the UM-Almadén secondary Hg isotopic standard, and the DORM-4 SRM are provided. UM = University of Michigan; UNCG = University of North Carolina Greensboro.

|  |  | |  |  |  | | |  | **∆^199^Hg (‰)/** |
| --- | --- | --- | --- | --- | --- | --- | --- | --- | --- |
| **Sample** | **δ^202^Hg (‰)** | ±**1SD** | **∆^201^Hg (‰)** | ±**1SD** | **∆^200^Hg (‰)** | ±**1SD** | **∆^199^Hg (‰)** | ±**1SD** | **∆^201^Hg (‰)** |
| UM-Almadén^1^ | -0.55 | 0.04 | -0.09 | 0.06 | 0.01 | 0.05 | -0.04 | 0.05 |  |
|  |  |  |  |  |  |  |  |  |  |
| SRM DORM-4 | +0.50 | 0.02 | +1.50 | 0.01 | +0.07 | 0.00 | +1.80 | 0.01 | 1.20 |
| DORM-4 Avg UM | +0.48 | 0.06 | +1.48 | 0.06 | +0.06 | 0.08 | +1.80 | 0.10 | 1.22 |
| UNCG Lot *N* = 7 |  |  |  |  |  |  |  |  |  |
|  |  |  |  |  |  |  |  |  |  |
| Sobreira de Cima Tomb I UE12 | -2.83 |  | +0.02 |  | +0.04 |  | +0.10 |  |  |
| Perdigões Tomb I Chamber #117 63 | -0.38 |  | -0.12 |  | -0.05 |  | -0.15 |  | 1.31 |
| Perdigões Tomb I Chamber B5 #59 97 | -1.22 |  | -0.07 |  | -0.02 |  | +0.07 |  |  |
|  |  |  |  |  |  |  |  |  |  |

^1^Average for isotope run only
